# The Impact of Structural Integration on Clinical Outcomes among Individuals with Serious Mental Illness and Chronic Illness

**DOI:** 10.1007/s10597-024-01293-4

**Published:** 2024-06-08

**Authors:** Elizabeth B. Matthews, Viktor Lushin, Eliza Macneal, Steve C. Marcus

**Affiliations:** 1 Graduate School of Social Service, Fordham University, 113 W. 60th St, New York, NY 10023 USA; 2 National Institutes of Mental Health, 6001 Executive Boulevard, MSC 9663, Bethesda, MD 20892-9663 USA; 3 Center for Mental Health, University of Pennsylvania, 3535 Market St, Philadelphia, PA 19104 USA

**Keywords:** Integrated care, Serious mental illness, Chronic disease, Community health centers

## Abstract

Though considered a best practice, there is substantial variation in how integrated behavioral health (IBH) services are structured. This study examined the impact of IBH structure on health outcomes among individuals with serious mental illness (SMI) and chronic disease receiving care in community health centers (CHCs). Data from the ADVANCE network identified 8,548 individuals with co-occurring SMI diabetes and 16,600 with an SMI and hypertension. Logistic regression tested whether IBH type impacted disease specific health outcomes among these populations. Among those with diabetes or hypertension, colocated care was associated with better health outcomes related to HbA1c, blood pressure control, and BMI compared to less coordinated and unintegrated care, though there was significant variation in this relationship across SMI diagnoses. Results reflect that colocation of primary care and behavioral health may improve outcomes for individuals with bipolar disorder or major depression and chronic disease, but that CHC-based integrated care may not be optimized for individuals with schizophrenia.

## Introduction

Individuals with serious mental illness (SMI), defined as a mental, behavioral, or emotional disorder that substantially interferes with one’s ability to function (Evans et al., 2016), experience disproportionately poor health outcomes and die an average of 10–20 years earlier than the general population, often because of untreated or undertreated chronic medical conditions (Colton & Manderscheid, 2006; Hjorthøj et al., 2017). Cardiovascular disease is the leading cause of death among this group (Colton & Manderscheid, 2006), and individuals with SMI are twice as likely to develop type II diabetes compared to the general population (Osborn et al., 2008). Similarly, estimates of cooccurring SMI and hypertension range from 35 to 61% (Murphy & Daumit, 2023; Onyeka et al., 2019; Pérez-Piñar et al., 2016; Rossom et al., 2022). Because of their complex psychiatric and medical needs, integrated behavioral health (IBH) care, where medical and mental health services are systematically coordinated, is a critical component of improving health outcomes for individuals with SMI and chronic disease (Bartels et al., 2018; Dregan et al., 2020; Schnitzer & Cather, 2021).

Despite its importance, integrated models of care have failed to consistently produce positive outcomes (Bradford et al., 2013; Chang et al., 2019; Cooper et al., 2013) for individuals with serious mental illness. The significant diversity with which integrated services are organized and clinically delivered may contribute to these inconsistent findings. At a basic, structural level, medical and behavioral health services can be *colocated*, or delivered in the same physical location, or *coordinated*, meaning delivered by community mental health and primary care partners that are formally affiliated, though physically separate (Heath et al.). A recent study within a single academic medical center found significant variation in whether individuals with serious mental illness utilized co-located or coordinated services, and that individuals utilizing co-located services had more medical and psychiatric admissions than those who received mental health treatment in specialty settings (Wetzler et al., 2020). While colocation of services is increasingly viewed as best practice, these findings raise important questions about which type of structural integration is most effective for individuals with SMI, particularly those with chronic health conditions. To better understand the association between structural integration of behavioral health and primary care services and clinical outcomes among this population, more research is needed to determine whether similar patterns persist across a larger sample of individuals and health settings, and whether individual level indicators, such as demographic characteristics and psychiatric diagnosis, may affect this relationship.

Community health centers (CHCs), safety net clinics that offer comprehensive care for low-income and underserved communities are an increasingly common setting for individuals with serious mental illness to receive mental health and primary care. A recent report by the Commonwealth fund and the African American Research Collaborative reports that behavioral health conditions are the common diagnoses treated in these settings, accounting for 40.7 million visits in 2022 alone (Horstman, 2024). Using a national sample of CHCs, this study generated specific information about how variation in the structure of IBH impacts clinical outcomes for individuals with SMI, specifically those with comorbid chronic health conditions, and whether these relationships vary by psychiatric diagnoses.

## Materials and Methods

This study used electronic health record (EHR) data from the Accelerating Data Value Across a National Community Health Center Network (ADVANCE) Clinical Research Network (CRN). The ADVANCE CRN is a member of PCORnet and led by OCHIN. As the nation’s most comprehensive set of health data from community-based health centers, ADVANCE integrates outpatient health record data and community-level data that represents over 9 million people seen in 236 community-based health systems across 39 states (OCHIN, 2023).

The present study utilized ADVANCE data from 2016 to 2021, the five most recent years available at the time of data extraction. We looked at each individual’s most recent episode of care, defined as their most recent 12 months of service utilization. Within this episode of care, our study population was limited to individuals who (1) were adults over the age of 18 (2) had an active diagnosis of SMI and a chronic health condition (diabetes or hypertension), and (3) utilized both mental health and primary care services within the ADVANCE CHC centers. Individuals were identified as having an active SMI if they had a primary care visit or mental visit associated with any of the following diagnoses: Bipolar Disorder (ICD10 codes F31x), Schizophrenia Spectrum Disorders (ICD10 Codes F20x, F22, and F25x) and/or Major Depression (ICD10 Codes F32.xx and F33.xx) within the sampled timeframe. We then limited the sample to those with Diabetes (Type I or Type II; ICD10 codes E10.9, E11x) and Hypertension (ICD10 code I10), which were the two most common chronic diseases among individuals with an SMI. Finally, because the present study focuses specifically on individuals receiving IBH within the ADVANCE network, we further restricted our sample to those who had at least one primary care visit and one mental health visit within their most recent episode of care. Primary care and mental health visits were identified using National Provider Identifier (NPI) based provider taxonomy codes and indicators of the specialization of the department where the visit occurred.

### Measures

Our primary independent variable of interest was the type of structural IBH (*colocated, coordinated*, and *unintegrated* care) utilized by CHC consumers with SMI. The ADVANCE database includes identifiers for each individual CHC site (i.e., physical location) and each CHC network (i.e., healthcare system), allowing the research team to identify mental health and primary care visits that occurred within the same CHC location (colocated care), within the same CHC network at different locations (coordinated care), and across different, unaffiliated CHC networks within the ADVANCE database (unintegrated care). Based on these indicators of visit location, each mental health visit was first designated as *colocated* if the individual also had a primary care visit at the same physical location within the previous 12 months. If the visit was not colocated, it was categorized as *coordinated* if the individual completed a primary care visit within the same CHC network but at the separate physical location within the previous 12 months. The visit was considered *unintegrated* if their physical health visits occurred at another physical location within another CHC network. We then created mutually exclusive categories that defined individuals as receiving either colocated, coordinated or unintegrated care based on which type of IBH they used the most during their 12-month episode of care.

We examined the impact of integration type (colocated, coordinated, unintegrated) on care quality and clinical outcomes. For each visit, ADVANCE data includes information related to vital statistics, prescribing, lab tests and procedures, and their results. Dependent variables related to clinical quality and outcomes for diabetes and hypertension care were based on established Healthcare Effectiveness Data and Information Set (HEDIS) metrics (Office of Disease Prevention and Health Promotion, nd) for both care quality (e.g., guideline concordant care) and clinical outcomes (e.g., improved health). Diabetes quality of care measures included dichotomous indicators of whether the individual had been prescribed blood pressure medication, had been prescribed diabetes medication, and whether the individual had their blood sugar levels tested in the past 12 months. Clinical outcome measures for individuals with diabetes included dichotomous indicators of whether the individual had any instance of controlled blood sugar (HbA1c < 8), blood pressure (< 140/90 mmHg), and body-mass index (BMI < 30) within their most recent 12 months of care. Quality of care for hypertension was represented by a dichotomous indicator of whether the individual had been prescribed blood pressure medication in their most recent 12 months of care. Clinical outcomes measures for hypertension care included dichotomous indicators of whether the individual any instance of controlled blood pressure (< 140/90 mmHg) or controlled BMI (< 30) in their most recent 12 months care.

Medical complexity was measured by the Charlson Comorbidity Index, an established scale that reflects medical comorbidity and mortality risk based on 19 medical conditions (Glasheen et al., 2019), with higher scores reflecting higher medical complexity. Scores were dichotomized using a median split in order to reflect differences between those with higher and lower levels of medical complexity within our sample. We also created binary indicators of social determinants that may impact health care utilization, including income above 100% of the poverty line, any reported instances of homelessness, or residing in a rural geographic area during their treatment episode. All study methods were reviewed and approved by the lead author’s Institutional Review Board.

### Analysis

Univariate statistics describe our study sample, stratified by chronic condition (diabetes and hypertension) and type of structural integration (colocated, coordinated and unintegrated). For each chronic condition, a series of ANOVAs explored differences in sample characteristics across integration types. Next, within each SMI diagnostic category (schizophrenia spectrum disorders, bipolar disorder, and major depressive disorder), logistic regression analyses were used to calculate the likelihood of receiving high quality care and experiencing good clinical outcomes for diabetes and hypertension, depending on whether the individual received *colocated, coordinated*, or *unintegrated* care. We conducted pairwise comparisons of these outcomes between groups of individuals receiving (1) colocated vs. coordinated care, (2) colocated vs. unintegrated care, and (3) coordinated vs. unintegrated care. Medical complexity, age, race/ethnicity, gender, housing status, income, and rurality were included as covariates in these models.

## Results

Sample demographics of individuals with SMI and cooccurring diabetes or hypertension are summarized in Table 1. There were significant differences in both psychiatric diagnoses and demographics of consumer populations across the three IBH types. Table 2 presents the adjusted odds ratios comparing quality and clinical outcome measures among individuals with SMI and diabetes across the three IBH groups (colocated, coordinated, and unintegrated), stratified by psychiatric diagnosis (schizophrenia spectrum, bipolar disorder and major depressive disorder). Compared to coordinated care, using colocated care was associated with increased odds of receiving an HbA1c test (AOR = 2.45, *p* < .05) among those with schizophrenia and an increased likelihood of having controlled blood sugar (AOR = 1.43, *p* < .001) for those with depression.

**Table 1:** Sample demographics of individuals with smi and chronic conditions

	Patients with SMI & Diabetes (*N* = 8,548)							Patients with SMI & Hypertension (*N* = 16,600)						
	Colocated *N* = 6,199		Coordinated *N* = 1,899		Unintegrated *N* = 460		p	Colocated *N* = 12,288		Coordinated *N* = 3,464		Unintegrated *N* = 848		p
	N	%	N	%	N	%		N	%	N	%	N	%	
**Age**							0.256							< 0.0001
18–40	1,009	16.28	314	16.62	69	15.00		1,764	14.36	580	16.74	124	14.62	
40–65	4,511	72.77	1,366	72.31	325	70.65		9,053	73.67	2,489	71.85	599	70.64	
65+	679	10.95	209	11.06	66	14.35		1,471	11.97	395	11.40	125	14.74	
**Race**							< 0.0001							< 0.0001
White	2,372	38.26	771	40.82	203	44.13		4,992	40.63	1,490	43.01	447	52.71	
Black	1,988	32.07	393	20.80	117	25.43		4,320	35.16	862	24.88	229	27.00	
Lantinx	1,227	19.79	485	25.67	74	16.09		1,840	14.97	732	21.13	83	9.79	
Other	283	4.57	136	7.20	49	10.65		458	3.73	184	5.31	67	7.90	
Unreported	329	5.31	104	5.51	17	3.7		678	5.52	196	1490	22	2.59	
**Gender**							< 0.0001							< 0.0001
Female	3,423	55.22	1,098	58.13	219	47.61		6,215	50.58	1,884	54.39	387	45.64	
Male	2,672	43.10	762	40.34	226	49.13		5,872	47.79	1,530	44.17	441	52.00	
Other	76	1.23	24	1.27	14	3.04		154	1.25	34	0.98	18	2.12	
Transgender	28	0.45	5	0.26	1	0.22		47	0.38	16	0.46	2	0.24	
**Homeless**	954	15.39	210	11.12	81	17.61	< 0.0001	2,282	18.57	419	12.10	155	18.28	< 0.0001
**Below 100% FPL** ^**+**^	4,465	72.03	1,336	70.73	416	90.43	< 0.0001	8,962	72.93	2,435	70.29	738	87.03	< 0.0001
**Rural Setting**	202	3.26	127	6.72	19	4.13	< 0.0001	439	3.57	250	7.22	32	3.77	< 0.0001
**High Medical Comorbidity**	4,566	73.66	1,324	70.09	362	78.70	< 0.0001	8,404	68.39	2,231	64.41	655	77.24	< 0.0001
**Diagnosis**														
Schizophrenia Spectrum	2,022	32.62	650	34.41	201	43.70	< 0.0001	3,641	29.63	1,037	29.94	366	43.16	< 0.0001
Bipolar Disorder	2,861	46.15	916	48.49	183	39.78	< 0.01	6,069	49.39	1,778	51.33	366	43.16	< 0.0001
Major Depression	2,409	38.86	675	35.73	146	31.74	< 0.0001	4,876	39.68	1,285	37.10	260	30.66	< 0.0001

*Note* All percentages are calculated based on total number of nonmissing cases; Mental health diagnoses are not mutually exclusive + FPL indicates Federal Poverty Level

**Table 2:** Clinical quality and health outcomes among individuals with smi and diabetes

	Colocated care		Coordinated care	Unintegrated care			Co-located vs. coordinated	Co-located vs. unintegrated		Coordinated vs. unintegrated		
	*N*	%	*N*	%	*N*	%	AOR^+^	AOR^+^		AOR^+^		
**Schizophrenia Spectrum Diagnosis (N=2,873)**	2,022	70.38	650	22.62	201	7.00						
*Quality Measures*												
Prescribed Blood Pressure Medication	1,008	49.85	328	50.46	91	45.27	0.91		1.28		1.32	
Prescribed Diabetes Medication	1,659	82.05	512	78.77	161	80.10	1.06		1.37		1.29	
HbA1c Draw	1,874	92.68	594	91.38	184	91.54	2.45*		1.08		0.38	
*Outcome Measures*												
HbA1c <= 8	1,289	71.23	380	62.29	129	73.71	1.14		0.98		0.98	
Blood Pressure < 140/90 mmHg	650	32.81	231	35.81	70	36.08	1.01		0.91		1.01	
BMI < 30	524	27.05	191	30.56	61	31.61	0.89		0.79		0.91	
**Bipolar Disorder Diagnosis (N=3,960)**	2,861	72.25	916	23.13	183	4.62						
*Quality Measures*												
Prescribed Blood Pressure Medication	1,440	50.33	475	51.86	86	46.99	0.86		1.11		1.27	
Prescribed Diabetes Medication	2,306	80.60	692	75.55	145	79.23	1.14		1.08		0.91	
HbA1c Draw	2,669	93.29	845	92.25	162	88.52	1.39		2.09		1.61	
*Outcome Measures*												
HbA1c <= 8	1,817	69.56	515	62.12	114	72.15	1.10		0.99		0.98	
Blood Pressure < 140/90 mmHg	984	34.98	339	37.88	69	38.98	0.95		0.93		0.89	
BMI < 30	661	23.87	194	21.82	45	26.01	1.11		0.98		0.81	
**Major Depressive Disorder Diagnosis (N=3,230)**	2,409	74.58	675	20.90	146	4.52						
*Quality Measures*												
Prescribed Blood Pressure Medication	1,311	54.42	364	53.93	77	52.74	0.98		1.04		1.08	
Prescribed Diabetes Medication	1,981	82.23	546	80.89	126	86.30	1.03		0.74		0.69	
HbA1c Draw	2,270	94.23	632	93.63	135	92.47	1.23		1.26		0.57	
*Outcome Measures*												
HbA1c <= 8	1,548	69.95	370	59.73	96	72.73	1.43**		1.00		0.82	
Blood Pressure < 140/90 mmHg	728	30.55	228	34.81	45	31.47	0.90*		1.00		1.08	
BMI < 30	639	27.23	161	24.88	42	30.00	1.17		1.10		1.14	

*Note* All percentages are calculated based on total number of nonmissing cases ^+^Adjusted odds Ratio; ^*^p < .05, ^**^p < .01, ^***^p < .001

Outcomes for individuals with SMI and hypertension are summarized in Table 3. Compared to coordinated services, colocated IBH utilization was associated with improved BMI control for those with bipolar disorder (AOR = 1.18, *p* < .01) and major depression (AOR = 1.18, *p* < .05), but not schizophrenia. Individuals with schizophrenia had lower odds of receiving blood pressure medication (AOR = 0.87, *p* < .05) but no more likely to receive higher quality care or achieve better health outcomes. Colocated, compared to unintegrated utilization was also associated with a higher likelihood of receiving blood pressure medication (AOR = 1.14, *p* < .001; AOR = 1.14, *p* < .001), and controlled BMI (AOR = 1.07, *p* < .001; AOR = 1.06, *p* < .001) among individuals with bipolar disorder and major depression, respectively. Individuals with bipolar disorder using colocated care were also more likely than the unintegrated group to have controlled blood pressure (AOR = 1.14, *p* < 001), though surprisingly individuals with depression were less likely to experience this outcome (AOR = 0.70, *p* < .001). No significant differences between colocated and unintegrated utilization were found among individuals with schizophrenia, though this group was more likely to receive a blood pressure medication prescription (AOR = 1.36, *p* < .05) when utilizing coordinated, rather than unintegrated care.

**Table 3:** Clinical quality and health outcomes among individuals with smi and hypertension

	Co-located care		Coordinated care		Unintegrated care		Co-located vs. coordinated		Co-located vs. unintegrated		Coordinated vs. unintegrated	
	*N*	%	*N*	%	*N*	%	AOR^+^		AOR^+^		AOR^+^	
**Schizophrenia Spectrum Diagnosis (N = 5044)**	3,641	72.18	1,037	20.56	366	7.26						
*Quality Measures*												
Prescribed Blood Pressure Medication	1,916	52.62	585	56.41	181	49.45	0.87*		1.25		1.36*	
*Outcome Measures*												
Blood Pressure < 140/90 mmHg	776	21.78	234	23.01	71	19.94	1.07		1.27		1.25	
BMI < 30	1,227	35.24	360	36.40	112	31.82	0.92		1.13		1.32	
**Bipolar Disorder Diagnosis (N = 8213)**	6,069	73.90	1,778	21.65	366	4.46						
*Quality Measures*												
Prescribed Blood Pressure Medication	3,319	54.69	975	54.84	190	51.91	0.99		1.14***		1.15	
*Outcome Measures*												
Blood Pressure < 140/90 mmHg	1,367	22.92	445	25.66	75	20.83	0.97		1.14***		1.11	
BMI < 30	1,902	32.28	486	28.35	104	29.97	1.18**		1.07***		0.96	
**Major Depressive Disorder Diagnosis (N = 6421)**	4,876	75.94	1,285	20.01	260	4.05						
*Quality Measures*												
Prescribed Blood Pressure Medication	2,753	56.46	759	59.07	139	53.46	0.89		1.14***		1.32	
*Outcome Measures*												
Blood Pressure < 140/90 mmHg	957	19.95	287	22.91	63	24.71	0.93		0.70***		0.78	
BMI < 30	1,571	33.24	353	28.52	87	34.94	1.18*		1.06***		0.99	

*Note* All percentages are calculated based on total number of nonmissing cases ^+^ Adjusted Odds Ratio; ^*^p<.05, ^**^p<.01, ^***^p<.00

## Discussion

The impact of IBH type on clinical outcomes appeared to differ by psychiatric diagnosis. Among individuals with comorbid diabetes and depression, colocated primary care and behavioral health appeared to improve blood sugar control better than coordinated models, but only for individuals with major depression. Similarly, colocation yielded better health outcomes than coordinated and unintegrated care on several metrics among individuals with bipolar disorder and major depression. In contrast, IBH utilization did not appear to impact outcomes among individuals with chronic medical conditions and schizophrenia spectrum disorder and had little impact on clinical quality as well.

Put together, these findings generally point to benefits associated with receiving colocated IBH for both overall service engagement and chronic disease management, but only among individuals with certain SMI diagnosis. In this sample, individuals with schizophrenia spectrum disorders did not benefit from CHC-based IBH in the same ways as other individuals with SMI. These differences may be partly driven by the complex nature of managing symptoms of psychosis, as both the health and mental health system persistently struggle to effectively engage individuals with schizophrenia spectrum disorders in care (Doyle et al., 2014; Kreyenbuhl et al., 2009; Tindall et al., 2018).

Alternatively, this may also point to the shortcomings of established models of primary care-based IBH. Several primary care-based integrated care models have been well-researched and widely disseminated in recent years, such as the Collaborative Chronic Care Model and the Primary Care Behavioral Health Model (Blackmore et al., 2018; Reed et al., 2016; Reist et al., 2022; Vogel et al., 2017). While these are often used to guide the development and implementation of IBH services, their evidence base is largely rooted in the management of acute depression and anxiety, rather than more complex and chronic mental health conditions. For example, in a recent meta-analysis of 78 studies examining the effectiveness of Chronic Collaborative Care programs, more than half focused on the treatment of depressive symptoms, while only 4 targeted bipolar disorder and none explicitly focused on other SMIs, like schizophrenia spectrum disorders (Woltmann et al., 2012). Although findings from this meta-analysis reflected consistent, positive effects of the Collaborative Care Model on symptom acuity and quality of life, these results may not be generalizable to all psychiatric conditions. Considered alongside the variation in positive health outcomes across clinical diagnoses identified in the present study, this indicates a need for more nuanced research that moves beyond questions related to the structural components of IBH to better understand the ‘active ingredients’ that shape how integrated services are clinically delivered (McGinty et al., 2021).

**Limitations.** Findings should be viewed in light of several limitations. First, our sample included only individuals who received their primary and behavioral health care in CHCs in the ADVANCE network, and we are therefore unable to explore outcomes among those who receive services in other community-based settings, or for consumers within our sample who may be receiving services elsewhere. This poses some limits on the generalizability our findings. Additionally, though the HEDIS measures used to define key outcomes are widely used indicators of health care quality and effectiveness, other important clinical outcomes, such as hospitalizations or medication adherence, were not available within the ADVANCE data, and may be an area for future research. Similarly, we focused only on two of the most common chronic conditions within our dataset; examination of the relationship between structural integration and health outcomes among those with other chronic diseases and among those with multiple comorbidities is therefore warranted. Further, additional exploration of the role of reverse causation, residual confounding are also areas for future study. Finally, while these results reflect differences in structural integration, or how services are physically organized, it does not account for the ways in which clinics vary in their clinical delivery of services.

## Conclusion

As the role of CHCs in delivering IBH to individuals with SMI continues to expand, best practices that can guide the delivery of high-quality care are needed. To our knowledge, the present study is the first to explore IBH for individuals with SMI within a national sample of CHCs and compare the impact of IBH structure on clinical outcomes among this group. Results highlight ways in which colocated, CHC-based IBH can yield positive outcomes for individuals with SMI and common comorbid health conditions.
